# Analysis of the amplified p-wave enables identification of patients with atrial fibrillation during sinus rhythm

**DOI:** 10.3389/fcvm.2023.1095931

**Published:** 2023-02-22

**Authors:** Taiyuan Huang, Patrick Schurr, Bjoern Muller-Edenborn, Nicolas Pilia, Louisa Mayer, Martin Eichenlaub, Juergen Allgeier, Marie Heidenreich, Christoph Ahlgrim, Marius Bohnen, Heiko Lehrmann, Dietmar Trenk, Franz-Josef Neumann, Dirk Westermann, Thomas Arentz, Amir Jadidi

**Affiliations:** Arrhythmia Division, Clinic for Cardiology and Angiology, University Heart Center Freiburg-Bad Krozingen, Faculty of Medicine, University of Freiburg, Freiburg im Breisgau, Germany

**Keywords:** atrial fibrillation, p-wave duration, electrocardiogram (ECG), diagnostic accuracy, atrial cardiomyopathy

## Abstract

**Aim:**

This study sought to develop and validate diagnostic models to identify individuals with atrial fibrillation (AF) using amplified sinus-p-wave analysis.

**Methods:**

A total of 1,492 patients (491 healthy controls, 499 with paroxysmal AF and 502 with persistent AF) underwent digital 12-lead-ECG recording during sinus rhythm. The patient cohort was divided into training and validation set in a 3:2 ratio. P-wave indices (PWI) including duration of standard p-wave (standard PWD; scale at 10 mm/mV, sweep speed at 25 mm/s) and amplified sinus-p-wave (APWD, scale at 60–120 mm/mV, sweep speed at 100 mm/s) and advanced inter-atrial block (aIAB) along with other clinical parameters were used to develop diagnostic models using logistic regression. Each model was developed from the training set and further tested in both training and validation sets for its diagnostic performance in identifying individuals with AF.

**Results:**

Compared to standard PWD (Reference model), which achieved an AUC of 0.637 and 0.632, for training and validation set, respectively, APWD (Basic model) importantly improved the accuracy to identify individuals with AF (AUC = 0.86 and 0.866). The PWI-based model combining APWD, aIAB and body surface area (BSA) further improved the diagnostic performance for AF (AUC = 0.892 and 0.885). The integrated model, which further combined left atrial diameter (LAD) with parameters of the PWI-based model, achieved optimal diagnostic performance (AUC = 0.916 and 0.902).

**Conclusion:**

Analysis of amplified p-wave during sinus rhythm allows identification of individuals with atrial fibrillation.

## Graphical Abstract

Illustration of PWD measurement and diagnostic performance from external validation. Panel **(A)** depicts the PWD measurement from the same ECG of an individual at standard scaling (10 mm/mV, 25 mm/s) and amplified scaling (60 mm/mV, 100 mm/s) using digital calipers. Panel **(B)** illustrates the ROC curves from external validation of all four diagnostic models regarding identifying individuals with AF, the component of each model and their AUC values are listed on the right margin. Panel **(C)** further delineates the diagnostic performance in external validation of each model in their respective optimal thresholds regarding accuracy, AUC, sensitivity, specificity, PPV and NPV. PWD, p-wave duration; ECG, electrocardiography; ROC curve, receiver operating characteristic curve; AF, atrial fibrillation; AUC, area under the curve; PPV, positive predictive value; NPV, negative predictive value. 
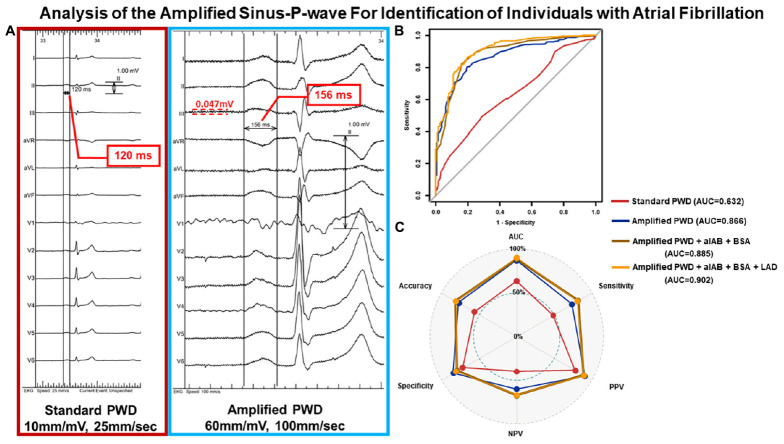

## Introduction

Atrial fibrillation (AF) is associated with significant morbidity and mortality (1). The high health care burden of AF and AF-related complications such as stroke or heart failure have prompted various attempts for risk prediction in the past decades, using ECG-derived p-wave indices (PWI) and cardiac imaging (2–4). Although several studies reported the potential predictive value of p-wave duration (PWD) for AF, ischemic stroke or mortality (5–8), the reported results were variable and the predictive value of PWD was limited, when measured using a standard scaling of 10 mm/mV, 25mm/s, i.e., standard PWD. In this context, we recently reported a novel p-wave analysis method that uses the measurement of p-wave duration (PWD) in amplified digital 12-lead-ECG (APWD) during sinus rhythm (SR), with high correlation to both the invasive bi-atrial activation time during electrophysiological study (EPS) and the extent of atrial fibrotic remodeling as detected by endocardial voltage and activation mapping in patients with atrial cardiomyopathy (9, 10). In the current study, we aim to compare the diagnostic performance of standard PWD to APWD and establish APWD-based diagnostic models for AF in a large cohort of consecutive patients.

## Materials and methods

### Study design and population

**FIGURE 1:**
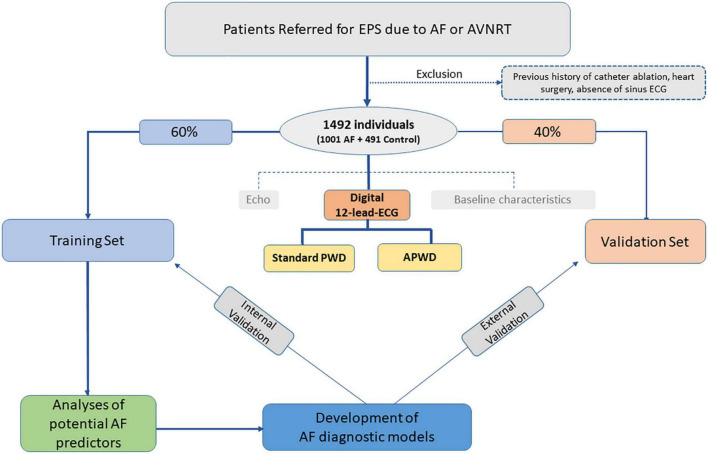
Study flowchart. EPS, electrophysiology study; ECG, electrocardiography; Echo, echocardiography; Standard PWD, duration of standard (non-amplified) p-wave; APWD, duration of amplified p-wave.

As illustrated in the study flowchart (Figure 1), Consecutive patients referred to our center between 2017 and 2021 for electrophysiological study were screened for study inclusion. Inclusion criteria were availability of a high-quality digital 12-lead ECG in sinus rhythm. Exclusion criteria were prior right- or left-atrial ablations, prior cardiac surgery or pacemaker-implantation of any kind. Patients with confirmed diagnosis of paroxysmal or persistent atrial fibrillation were allocated to the AF-cohort. Patients who presented with AF in their admission ECG, first underwent electrical cardioversion to sinus rhythm and were scheduled for pulmonary vein isolation (PVI) 6–8 weeks thereafter. In these patients, the analysis of 12-lead-ECGs during sinus rhythm was based on ECG recordings from the rehopsitalisation (i.e., 6–8 weeks after electrical cardioversion to SR). For the purpose of the current study, patients diagnosed with atrio-ventricular nodal reentrant tachycardia in the absence of AF or other arrhythmia were considered as control cohort.

### Training and validation sets

All individuals were subsequently randomized into training and validation set with predefined ratio of 3:2. The former was used to develop diagnostic models for AF and internally validate model performance, the latter was used to validate model performance in an external way. Current study conforms to the principles outlined in the Declaration of Helsinki and was approved by the institutional ethics committee, all patients provided written informed consent prior to enrollment.

### Digital 12-lead-ECG recording and p-wave analysis

**FIGURE 2:**
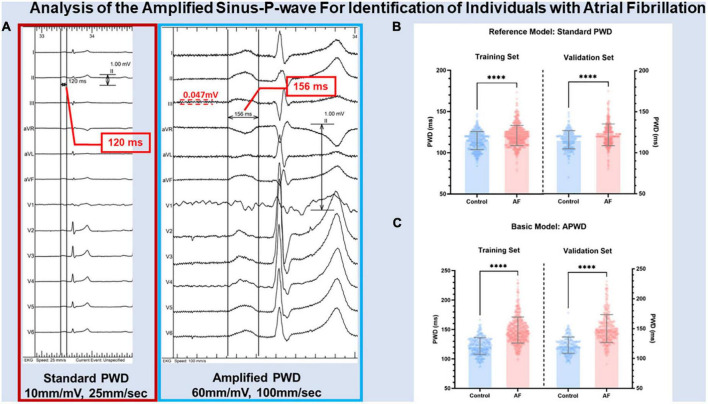
Illustration of PWD measurement in standard and amplified scaling. Panel **(A)** illustrates the results of p-wave duration from the same digital 12-lead ECG measured at standard scaling (10 mm/mV, 25 mm/s) and amplified scaling (60 mm/mV, 100 mm/s) using digital calipers. PWD was measured from the earliest p-wave onset until latest p-wave ending in any of the 12 leads at respective scaling. The noise level of annotated by the red dashed lines. Panel **(B,C)** illustrate the difference between control and AF cohorts in internal and external validation using standard PWD (reference model) and APWD (basic model), respectively. PWD, p-wave duration; standard PWD, duration of standard p-wave; APWD, duration of amplified p-wave; ^****^p < 0.001.

Digital 12-lead-ECG was recorded during sinus rhythm in all study patients using LabsystemPro EP-system (Boston Scientific) prior to sedation at the beginning of electrophysiology study with the following filter settings: 0.05–100 Hz without additional 50 Hz filtering at a sampling rate of 1,000 Hz. The duration of the standard p-wave (standard PWD) was measured at 10 mm/mV and 25 mm/s scaling and the duration of amplified p-wave (APWD) was measured at amplified scaling (60–120 mm/mV and 100 mm/s) (Figure 2A and Supplementary Figure 1). The duration of p-wave was determined using digital calipers from the earliest p-wave onset until latest p-wave ending in any of the 12 leads. Standard PWD and APWD were calculated as the mean value of three consecutive beats measurements. Advanced inter-atrial block (aIAB) was defined as initially positive p-wave with negative terminal deflection in two of three inferior leads. The measurement of standard PWD, APWD and aIAB was performed independently by two cardiologists who were blind to patients’ clinical characteristics.

## Statistical analysis

Continuous variables were expressed as mean ± SD or median ± interquartile range based upon distribution status. Given the sample size of our study, the normality test was performed using both Shapiro–Wilk’s test and visual estimation of the P–P plot. The homoscedasticity of the dataset was performed using Levene’s test. Based on the results of normality and homoscedasticity, comparisons between two cohorts was performed using t-test or Mann-Whitney U test. Categorical variables were expressed as frequency and percentage (%) and were compared by Chi-square test or Fisher’s exact test. Inter-and intra-observer variability was analyzed using intra-class correlation coefficient (ICC), Bland–Altman plot, and correlation curve were used to illustrate the consistency in PWD measurement within the same observer and between observers.

### Optimal PWD parameter selection

**FIGURE 3:**
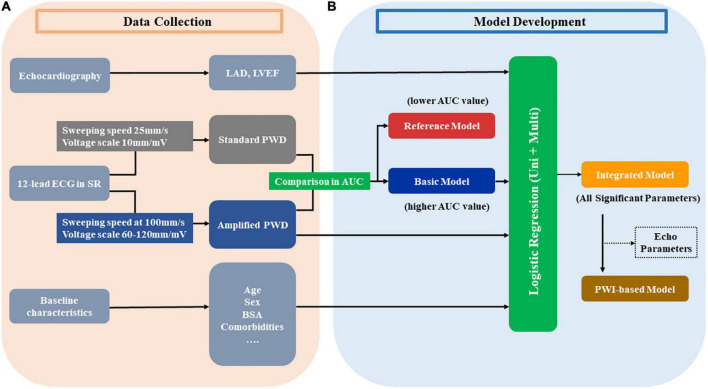
Development of diagnostic models from the training set. Panel **(A)** illustrates the data collection in the training set in the current study, different parameters from echocardiography, 12-lead ECG and other baseline characteristics are collected. Every ECG was measured at both standard setting (25 mm/s, 10 mm/mV) and amplified setting (100 mm/s, 60–120 mm/mV) to acquire the standard PWD and amplified PWD, respectively. Panel **(B)** depicts the steps in developing models. Amplified PWD and standard PWD were compared regarding their AUC in discriminative power to identify patients with AF. The one with higher AUC value is used as the basic model and proceed to further steps whereas the one with lower AUC value is used as the reference model. Basic model, along with other echo and baseline parameters are selected by univariate and subsequent multivariate parameters. The integrated model consists of all the significant parameters from the logistic analysis. Additionally, in order to create an alternative model with less variables and more oriented at ECG parameters, a PWI-based model is developed by excluding echo parameters (if any) from the integrated model. AUC, area under the curve; BSA, body surface area; ECG, electrocardiography; Echo, echocardiography; LAD, left atrial diameter; LVEF, left ventricular ejection fraction; PWD, p-wave duration; PWI, p-wave index; Uni, univariate; Multi, multivariate.

As illustrated in Figures 2B, C, 3A, the current study provided both standard PWD and APWD as candidate PWD parameters for model development. By comparing their efficacy in identifying individuals with AF, the one with superior discriminatory performance (AUC or C-index) would be used as a basic model and undergo further steps for multivariable AF diagnostic model construction whereas the other would be used as a reference model.

### Model development

Model development was performed in the training set (Figure 3B). The optimal PWD parameter, along with other variables describing baseline characteristics were used as candidate variables prior to univariable logistic regression analysis. Subsequently, significant variables (*p* < 0.05) in univariable regression would undergo further multivariable regression analysis. As a result, variables that maintained *p* < 0.05 after multivariable logistic regression would be selected to develop the multivariable AF diagnostic model (Integrated model). Additionally, alternative diagnostic models were also proposed using less variables in order to improve model practicability and test model stability with regard to diagnostic efficacy.

### Model validation

Validation of models were performed both internally (in training set) and externally (in validation set) regarding their efficacy in discrimination, calibration, net benefit and diagnostic accuracy using optimal thresholds.

Discriminatory power of each model for identification of AF patients was quantified by area under the curve (AUC) of respective receiver operating characteristics (ROC) curve, ranging from 0.5 (random forecast) to 1.0 (perfect discrimination). Additional comparison regarding discriminatory power between models was performed using integrated discrimination improvement (IDI). Two-tailed *p* values were calculated for all tests and considered significant at *p* value < 0.05.

After the components of each model were determined, the individual probability for AF by each model was estimated. Calibration plot of each model was created to visualize the agreement between estimated probabilities for AF and the actual probabilities observed in each set. Moreover, Brier score, as a parameter that quantifies the accuracy of probability by diagnostic model (0 for total accuracy, 1 for wholly inaccurate) was calculated and noted in the calibration curves.

The net benefit in clinical usefulness of selected models across a range of probability threshold was illustrated by decision curve analysis (DCA). The ‘None’ and ‘All’ curve indicated the expected net benefit when intervention was performed to “none” and “all” of the patients.

Diagnostic performance evaluation of each model consisted of sensitivity, specificity, positive predictive value (PPV), negative predictive value (NPV) and accuracy. Based on ROC curve coordinates of each model from training set, optimal probability threshold from every model for AF was determined by Youden Index (sensitivity + specificity − 1). Diagnostic performance of each model was subsequently evaluated using determined probability thresholds in both training and validation sets.

Statistical analysis was performed with SPSS version 27.0 for Macintosh (IBM-Corporation, Armonk, NY, USA), GraphPadPrism-V9.0 for Macintosh (GraphPad Software, LaJolla, CA, USA) and R software version 4.0.3^1^ with rms, pROC, ggplot2, rmda, ggDCA, caret, and PredictABEL packages.

## Results

### Patient characteristics and randomization

A total of 1,492 individuals were included: 491 (32.9%) patients with AVNRT but no history of AF or other arrhythmias were in the control cohort, and 1,001 (67.1%) patients in the AF cohort (499 (33.5%) with paroxysmal AF and 502 (33.6%) with persistent AF). Baseline characteristics are presented in Table 1 and Supplementary Table 1. Patients with AF were predominantly male, had higher body mass index (BMI), larger body surface area (BSA), larger LA-diameters (LAD), lower LVEF, presented more often hypertension, stroke and coronary artery disease. Subsequently, 896 (60.1%) of the total patients were randomized into training set and 596 (39.9%) patients into validation set (Figure 1). No significant differences in baseline characteristics were observed between training and validation set (Supplementary Table 2).

**TABLE 1:** Baseline characteristics of total cohort.

Variables	Overall (*n* = 1492)	Control (*n* = 491)	AF (*n* = 1001)	*P*-value
Age, years	60.14 ± 14.42	60.02 ± 17.14	60.19 ± 12.88	0.828
Female, *n* (%)	631 (42.30%)	262 (53.40%)	369 (36.90%)	<0.001
Paroxysmal AF, *n* (%)	499 (33.44%)	0	499 (49.85%)	<0.001
BMI, kg/m^2^	27.09 ± 4.66	25.72 ± 4.39	27.75 ± 4.64	<0.001
BSA, cm^2^	1.97 ± 0.23	1.87 ± 0.21	2.01 ± 0.22	<0.001
LAD, mm	40.19 ± 6.23	36.55 ± 4.95	41.90 ± 6.03	<0.001
LVEF,%	59.81 ± 9.87	61.67 ± 8.76	58.92 ± 10.25	<0.001
Hypertension, *n* (%)	778 (52.10%)	212 (43.20%)	566 (56.5%)	<0.001
Diabetes, *n* (%)	127 (8.50%)	35 (7.10%)	92 (9.2%)	0.180
Stroke, *n* (%)	36 (2.40%)	4 (0.8%)	32 (3.2%)	0.004
TIA, *n* (%)	36 (2.40%)	10 (2.0%)	26 (2.6%)	0.593
CHD, *n* (%)	177 (11.90%)	35 (7.1%)	142 (14.20%)	<0.001
CHA2DS2-VASc score	1.94 ± 1.45	1.74 ± 1.47	2.03 ± 1.43	<0.001
GFR (ml/min/1.73 m^2^)	80.58 ± 19.79	84.24 ± 19.71	78.79 ± 19.59	<0.001
Creatinin clearance (mg/dl)	0.96 ± 0.33	0.89 ± 0.21	0.99 ± 0.38	<0.001
Antiarrhythmia drugs, *n* (%)	522 (35.0%)	0	522 (52.1%)	<0.001
Amiodarone, *n* (%)	247 (16.60%)	0	247 (24.7%)	<0.001
Dronedarone, *n* (%)	14 (0.9%)	0	14 (1.4%)	0.019
Flecanid, *n* (%)	205 (13.7%)	0	205 (20.5%)	<0.001
Propafenon, *n* (%)	11 (0.7%)	0	11 (1.1%)	0.044
Sotalol, *n* (%)	45 (3.0%)	0	45 (4.5%)	<0.001
Anticoagulant, *n* (%)	940 (63.0%)	0	940 (93.9%)	<0.001
VKA, *n* (%)	159 (10.7%)	0	159 (15.9%)	<0.001
Apixaban, *n* (%)	200 (13.4%)	0	200 (20.0%)	<0.001
Rivaroxaban, *n* (%)	429 (28.8%)	0	429 (42.9%)	<0.001
Edoxaban, *n* (%)	63 (4.2%)	0	63 (6.3%)	<0.001
Dabigatran, *n* (%)	89 (6.0%)	0	89 (8.9%)	<0.001

AF, atrial fibrillation; BMI, body mass index; BSA, body surface area; LAD, left atrial diameter; LVEF, left ventricular ejection fraction; TIA, transient ischemic attack; CHD, coronary heart disease; GFR, glomerular filtration rate; VKA, vitamin-K antagonist.

### Differences between “standard PWD” and “APWD” in control cohort vs. AF cohort

As illustrated in Figures 2B, C, both standard PWD and APWD differed significantly in training set between control and AF cohort (standard PWD: 115 ± 11 ms in control cohort vs. 121 ± 12 ms in AF cohort, *p* < 0.001; APWD: 122 ± 14 ms in control cohort vs. 149 ± 22 ms in AF cohort, *p* < 0.001) while the difference was more pronounced in the latter. Consistent findings were observed in the validation set, in contrast to standard PWD (116 ± 11 ms in control cohort vs. 122 ± 13 ms in AF cohort, *p* < 0.001), APWD displayed larger differences between two cohorts (122 ± 14 ms in control cohort vs. 150 ± 23 ms in AF cohort, *p* < 0.001). Subgroup sex-specific analyses revealed consistency of these findings (Supplementary Figure 2 and Supplementary Table 3). Subsequently, sensitivity analyses were performed to exclude the potential bias mediated by use of antiarrhythmia pharmaceuticals and anticoagulants in the AF cohort. As listed in Table 1, 52.1% of patients in AF cohort had current or history use of antiarrhythmia drugs (Amiodarone/Dronedarone/Flecanid/Propafenon/Sotalol) within four weeks that might influence the atrial de- and repolarization. In those without use of aforementioned drugs, comparisons in APWD and standard PWD were performed between control and AF cohort. As a result, APWD was significantly longer in the AF cohort than in the control cohort (143.9 ± 22.4 ms vs. 122.9 ± 14.8 ms, *p* < 0.001). In standard PWD, on the other hand, although the difference between two cohorts reached statistical significance (*p* < 0.001), the absolute value was insufficient to provide clinical implication (119.4 ± 12.3 ms vs. 115.7 ± 11.1 ms). Moreover, oral anticoagulants (OAC) were used in over 90% of AF cohort, in the remaining 61 OAC-free patients in AF cohort and 491 patients in control cohort, a profound difference in APWD remained significant (144.1 ± 24.2 ms vs. 122.9 ± 14.8 ms, *p* < 0.001). Similar findings were also observed in standard PWD with marginal absolute difference (120.3 ± 12.9 ms vs. 115.7 ± 11.1 ms, *p* = 0.004).

### Reproducibility in measurements using amplified p-wave analysis

Among 1,492 study patients, 25 (1.7%) presented sinus ECG recording with unsatisfying noise level (baseline noise above 0.08 mV). In those cases, the digitalized 12-lead-ECGs that were recorded within the 3-month preceding the EPS were taken for analysis. Each 12-lead digitalized ECG was measured by two independent cardiologists using digital calipers. The amplified scaling of each ECG was manually adjusted to obtain an optimal signal-to-ratio that allowed visualization of the entire p-wave (Supplementary Figure 1). As a result, among 1492 ECG in total cohort, 83.3% of them were measured at an amplified scaling of 60 mm/mV, 100 mm/s, and the remaining 16.7% of cases, due to low p-wave amplitudes, were measured at 120 mms/mV, 100 mm/s. An excellent agreement was observed both in intra-observer (ICC 0.951, 95%CI: 0.936–0.963) and inter-observer (ICC 0.915, 95%CI: 0.875–0.941) measurements. Both the intra- and inter-observer measurements were performed on the same p-waves, but after a three-month time interval between the first and second measurement. Bland-Altman plots and correlation curves illustrate the agreement in each measurement within and between observers (Supplementary Figure 3).

### Discriminatory performance of standard PWD and APWD to identify individuals with AF

In order to determine the optimal candidate between standard PWD and APWD for further development of diagnostic models, C-index from training set was calculated to compare the discriminatory power between standard PWD and APWD. In contrast to standard PWD (C-index: 0.637, 95%CI: 0.599–0.675), APWD achieved significantly higher C-index value (0.86, 95%CI: 0.836–0.884, *p* < 0.001). Consistent results were observed in validation set (0.632, 95%CI: 0.586–0.679 in standard PWD vs. 0.866, 95%CI: 0.836–0.895 in APWD; *p* < 0.001).

### Univariable and multivariable analysis for variable selection (training set)

Given the significant superiority in discriminatory power of APWD over standard PWD between control and AF cohort, APWD instead of standard PWD was used for the construction of further AF diagnostic models. As shown in Table 2, in univariable analysis, significant AF predictors (*p* < 0.05) were further included in multivariable logistic regression analysis. As a result, only APWD (*p* < 0.001), BSA (*p* = 0.034), and LAD (*p* = 0.008) remained significant, and were further incorporated for identification of individuals with AF.

**TABLE 2:** Univariable and multivariable analysis of AF predictors.

Univariable	*P*-value	OR	95%CI	
aIAB	0.996	9.87e + 08	0	–
Sex	<0.001	0.535	0.404	0.709
Age	0.521	1.003	0.994	1.012
APWD	<0.001	1.096	1.082	1.11
Hypertension	<0.001	2.601	1.951	3.468
Diabetes	0.223	1.382	0.821	2.327
Stroke	0.018	11.286	1.514	84.139
TIA	0.529	1.393	0.497	3.904
CHD	<0.001	2.705	1.597	4.582
BMI	<0.001	1.104	1.068	1.142
BSA	<0.001	14.415	7.138	29.109
LAD	<0.001	1.207	1.168	1.247
LVEF	<0.001	0.964	0.949	0.979
**Multivariable**	***P*** **-value**	**OR**	**95%CI**	
Sex	0.236	0.78	0.517	1.177
APWD	<0.001	1.087	1.071	1.103
Hypertension	0.715	0.927	0.616	1.394
Stroke	0.084	8.803	0.747	103.71
CHD	0.529	1.249	0.626	2.492
BMI	0.612	0.986	0.935	1.04
BSA	0.034	3.53	1.103	11.293
LAD	0.008	1.056	1.015	1.1
LVEF	0.841	1.002	0.981	1.024

aIAB, advanced inter-atrial block; APWD, duration of amplified p-wave; TIA, transient ischemic attack; CHD, coronary heart disease; BSA, body surface area; BMI, body mass index; LAD, left atrial diameter; LVEF, left ventricular ejection fraction.

### Development of diagnostic models for AF (training set)

As described under supplemental statistical section, standard PWD was therefore used as a reference model. Given the above-mentioned results, APWD, BSA and LAD were considered for integrated model construction. In order to facilitate model practicability in clinical setting, we intended to provide two alternative models with less variables: (1) APWD alone was chosen as a basic model. (2) A PWI-based model was established as another alternative ECG model. In this context, advanced Inter-atrial block (aIAB), as a valuable predictor of left atrial arrhythmogenic/fibrotic substrate with high specificity (9), was incorporated to the models. As a result, we developed four diagnostic models for identification of individuals with AF: (1) Reference model (standard PWD), (2) Basic model (APWD), (3) PWI-based model (APWD + aIAB + BSA), and (4) Integrated model (APWD + aIAB + BSA + LAD).

### Validation of diagnostic models for AF

#### Discrimination between control and AF cohort

**FIGURE 4:**
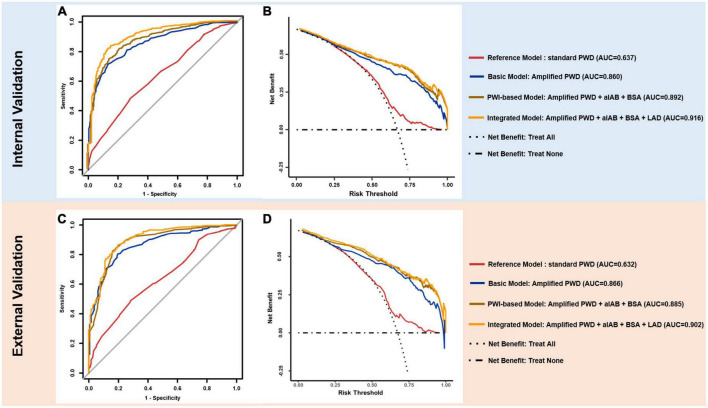
ROC and DCA curves. In internal (upper panel) and external validation (lower panel), ROC curves of reference model (red), basic model (blue), PWI-based model (brown) and integrated model (yellow) are plotted **(A,C)** with model components and AUC value annotated at right side. DCA **(B,D)** illustrates the clinical impact (net benefit) of diagnostic model with reference curves of *“Treat All”* and *“Treat None”*. *“Treat All”* and *“Treat None”* described the impact of intervention for *“All”* and *“None”* of individuals for target outcome (AF) respectively, when diagnostic model is not applied. ROC, receiver operating characteristics; AUC, area under the curve; DCA, decision curve analysis; PWD, p-wave duration.

As illustrated in Figures 4A, C, the integrated model achieved optimal discriminatory performance in both internal (AUC 0.916) and external validation (AUC 0.902) in comparison to the basic model and the PWI-based model, indicating its prominent potential for identification of AF patients. Although alternative models contained less variables, they still maintained an AUC value over 0.85 in both validations, suggesting that APWD was an essential component for identification of AF patients (Supplementary Table 4). In contrast, standard PWD achieved significantly lower discriminatory performance (AUC: 0.637 and 0.632). Additionally, we performed a subgroup analyses to evaluate the discriminative performance of APWD and standard PWD in differentiation between paroxysmal AF cohort from control cohort. In the training set, APWD achieved an AUC of 0.777 (95%CI: 0.740–0.813) whereas standard PWD achieved only mild discriminative power (AUC: 0.624, 95%CI: 0.579–0.668). Similar results were observed in the validation set regarding AUC between paroxysmal AF cohort and control cohort (APWD: 0.780, 95%CI: 0.734–0.826 vs. Standard PWD: 0.623, 95%CI: 0.568–0.678). Integrated discrimination improvement (IDI) is a statistical parameter to evaluate the ability of a model to improve the average sensitivity without reducing average specificity. As shown in Supplementary Table 5, both PWI-based model and integrated model showed significantly improved discriminatory performance compared to the basic model in internal and external validation. Based on AUC comparison between integrated- and PWI-based model (*p* < 0.001 and *p* = 0.005 in internal and external validation) and IDI value, the integrated model was associated with higher accuracy to correctly identify patients with AF than PWI-based model.

#### Calibration between estimated and observed AF probabilities

Brier score, which is defined as the mean squared difference between the observed and estimated outcome, allows estimation of model calibration performance (“0” for optimal calibration, “1” for entirely inaccurate). As illustrated in calibration curves (Supplementary Figure 4) integrated model displayed excellent agreement between estimated and observed AF probability with Brier score of 0.103 and 0.112 in internal and external validation, respectively. PWI-based Reference model and basic model, despite fewer variables, also demonstrated rather good agreement between estimated and observed AF probability. Reference model, in contrast to other APWD-based models, achieved insufficient performance with Brier score of 0.209 and 0.210 in respective validation.

#### Decision curve analysis for net benefit assessment

As illustrated in Figures 4B, D, the net benefit for clinical usefulness by each diagnostic model across a range of AF-risk thresholds was assessed using Decision curve analysis (DCA). Results from internal and external validations demonstrated comparable promising net benefit across potential thresholds by integrated and PWI-based models, indicating their robust efficacy in identification of AF patients. The basic model, on the other hand, presented slightly reduced net benefit in comparison to integrated model and PWI-based model when thresholds were above 0.50 in both internal and external validation. In contrast, the reference model (standard PWD) demonstrated only marginal benefit in both internal and external validation, making only marginal difference than treating all or none of individuals when no diagnostic model was used.

### Diagnostic performance using optimal thresholds and development of nomograms for identification of AF patients

**FIGURE 5:**
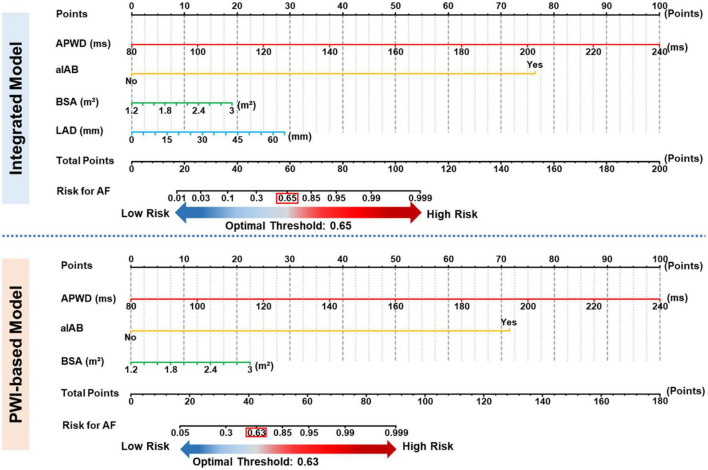
Nomograms for identification of AF patients. Nomogram of PWI-based model **(upper panel)** and Integrated model **(lower panel)**. Each value from respective scale corresponds to a specific value at the top points scale, and the total points correspond to the estimated risk **(bottom scale)** for AF by respective model. APWD, duration of amplified p-wave; aIAB, advanced inter-atrial block; BSA, body surface area; LAD, left atrial diameter.

Based on the ROC curve of each model in training set, respective optimal thresholds were determined and subsequently applied in both training and validation sets to dichotomize the AF probability as high risk (above threshold) or low risk (below threshold). As a result, the optimal thresholds of the reference model and basic model were determined with a standard PWD of 121 ms and APWD of 136 ms, respectively. The optimal thresholds of PWI-based model and integrated model, however, due to their multi-variable feature, were determined by ROC curves based on their estimated AF probability. After calculating Youden index, we determined AF probability of 0.63 and 0.65 as optimal thresholds for the PWI-based model and the integrated model, respectively. Detailed diagnostic performance of each model in internal and external validation was illustrated in Supplementary Figure 5 and Table 3. In an additional effort to facilitate the application of PWI-based and integrated model for identification of patients with AF, we developed a nomogram for each of those two models and incorporated the optimal thresholds to assist further decision-making (Figure 5), each value in the listed parameters (APWD, aIAB, BSA, etc.) can be converted into a corresponding points at the ‘Points scale’ at the top, and the sum of all points from every parameter can be used to estimate the risk for AF. Based on the individual result of APWD, IAB, BSA (and LAD), the nomogram allowed estimation of the personalized risk for AF, and by comparing it with the ROC-defined optimal threshold, each individual would be assigned as either high or low risk for AF (illustrative example in Supplementary Figure 6 to guide the use of both nomograms).

**TABLE 3:** Optimal thresholds in models with diagnostic performance.

Randomization	Model	Reference	Basic	PWI-based	Integrated
	**Component**	**Standard PWD**	**APWD**	**APWD, aIAB, BSA**	**APWD, aIAB, BSA, LAD**
	**Threshold**	**121 ms**	**136 ms**	**AF probability 0.63**	**AF probability 0.65**
Training set	Accuracy	55.7%	76.6%	81.4%	81.7%
	sensitivity	47.8%	71.5%	81.1%	81.1%
	specificity	72.1%	86.9%	81.8%	82.8%
	PPV	77.5%	91.0%	90.0%	90.5%
	NPV	40.5%	60.3%	68.3%	68.5%
Validation set	Accuracy	56.0%	76.8%	80.9%	80.7%
	sensitivity	48.5%	73.4%	81.8%	80.9%
	specificity	71.7%	84.0%	78.9%	80.4%
	PPV	78.0%	90.5%	88.9%	89.5%
	NPV	40.2%	60.4%	67.7%	67.0%

Standard PWD, duration of standard (non-amplified) p-wave; APWD, duration of amplified p-wave; aIAB, advanced inter-atrial block; BSA, body surface area; LAD, left atrial diameter; PPV, positive predictive value; NPV, negative predictive value.

## Discussion

The present study provides three main findings: (1) Compared to standard PWD, the diagnostic models based on APWD significantly improve the accuracy for the identification of patients with AF. (2) Integration of APWD with IAB, and BSA allowed development of a multi-variable PWI-based model with optimal performance for identification of patients with AF. (3) Addition of echocardiographic left atrial diameter to the PWI-based model further improved the diagnostic power for AF.

### Previously described diagnostic tools for atrial fibrillation

Pathological mechanisms responsible for AF development and progression are intertwined and triggered by multiple factors including stretch-induced fibrosis, fatty infiltration, myocardial inflammation, heterogeneous conduction, etc. (1, 11, 12). Previous studies proposed several predictive scores for new-onset AF based on various risk factors: The C2HEST score consists of comorbidities that predict 1-year risk for AF with C-index of 0.734 (13). Other models including CHARGE-AF score and FHS score reach C-index of 0.77 and 0.78 for 5- and 10-year AF risk, respectively (14, 15). Nevertheless, the C-index reported from those studies indicated moderate accuracies. In addition, the complexity and high number of risk factors that are mandatory in those scores also limit the practicability in clinical practice. Therefore, ECG-analysis has been favored with its advantages of being non-invasive and cost effective.

In the past decades, important efforts have been made in various studies to determine the ideal ECG-parameter for AF prediction. PWI including p-wave dispersion, p-wave axis, p-wave duration, P-terminal force in V1, p-wave morphological criteria and other parameters have been introduced and assessed for their diagnostic value for predicting AF or cardiovascular mortality (3, 5, 6, 16). Nevertheless, controversies still remain as the predictive accuracy was not always encouraging among studies. Nielsen et al. analyzed the standard PWD of more than 285,000 individuals from Copenhagen ECG study, and reported that individuals with very short PWD (<89 ms) and very long PWD (>130 ms) have a respective hazard ratio of 1.6 and 2.06 for incident AF in comparison to individuals with a PWD between 100 and 105 ms (7). They stated the hypothesis that a more rapid conduction time might provide a substrate for reentry in early stages of arrhythmias. However, in our current study, short APWD <90 ms was only observed in individuals without AF. Conte et al. reported a threshold of 121 ms to differentiate between paroxysmal AF patients from healthy individual with an AUC of 0.80, however, the reported sensitivity was only 63% and the total sample size was 76 individuals only (17). Our study confirmed their findings in a larger cohort, regarding the threshold of 121ms. However, the diagnostic value of standard PWD in our larger cohort is limited with an AUC of 0.63. In the current study, an APWD > 136 ms, was found to have a greater potential to identify patients with AF than PWD.

### Relationship of APWD with atrial cardiomyopathy and risk of AF

We recently reported that the duration of the digitally recorded, highly amplified sinus-p-wave (APWD) accurately represents both the invasively measured bi-atrial activation time and the extent of atrial low voltage areas (as a electrophysiological marker of atrial cardiomyopathy), thus allowing identification of AF patients with advanced atrial cardiomyopathy, who are at risk for recurrent AF after catheter ablation therapy (10). In contrast to APWD, the standard p-wave duration (10 mm/mV and 25 mm/s) may underestimate the atrial conduction time (9). This is even more pronounced in individuals with advanced atrial fibrotic cardiomyopathy who present reduced p-wave voltages (due to the loss of synchronously depolarized atrial cardiomyocytes). Thereby, the standard PWD does not allow accurate measurement of the true atrial conduction time, leading to an insufficient diagnostic performance to identify individuals with AF.

### Rationale for developing alternative diagnostic models using APWD

Previous studies focusing on development of prediction model predominantly aimed to propose one model with optimal performance by incorporation of multiple variables. FHS score required the information of eight variables and CHARGE-AF score demanded data of more than ten variables to predict new-onset AF (14, 15). Albeit they were developed from large data cohorts and enabled long-term risk estimation, the complexity of models inevitably limited their application in real-world practice. In the current study, in an aim to further improve its practicability, we proposed alternative models with even fewer variables while maintaining a rather comparable diagnostic efficacy. As APWD alone already displayed robust superiority in discriminatory performance (AUC over 0.85), it would be rational to be a basic model. Furthermore, among LAD, aIAB, and BSA, construction of another alternative model by different combinations with APWD should take into account both the strength and weakness of each variable. BSA, is easily available, as it is calculated from patient’s height and weight. AIAB was shown to be predictive for AF-associated atrial cardiomyopathy and AF development (9, 18). We therefore combined it to APWD in the PWI-based and integrated models, leading to an improved identification of AF patients. In the current study, LAD was routinely measured in echocardiography in all patients. Although it assesses the LA size in one direction only, it could slightly improve identification of AF patients in our AF models. However, we expect that integration of LA volume (as a 3D parameter of LA size) and/or LA strain would further improve the diagnostic models for AF. In this context, a diagnostic model (PWI-based model) without LAD but focused on APWD and AIAB can be considered as an alternative model with high diagnostic performance for detection of AF patients (AUC: 0.892).

### Clinical potential in APWD-based models

The current study is the first to use amplified p-wave analysis during sinus rhythm and reveals that APWD alone or in combination with a few other predictors is of great potential in differentiating individuals with AF from those without. Thereby, the new models identify the *current* predisposition for AF and provide the option for targeted screening of individuals at risk for AF, instead of a non-selective population-wide screening. Individuals that are identified as high risk for AF using current models may benefit from a more frequent ECG monitoring for AF.

### Artificial intelligence (AI) and ECG-analysis for AF detection

Recently, AI-empowered algorithms were reported to facilitate AF-screening using the newest generation of portable ECG-devices. These devices and algorithms allow direct detection of AF occurrence based on RR-interval analysis (19). Nevertheless, detection of short self-limited episodes of AF may lead to a test and treatment cascades affecting the individuals’ quality of lives and questioning whether the use of single-lead ECG devices is suited for AF screening at population level without prior risk stratification for underlying cardiovascular diseases (20). The combination of our current diagnostic models for AF (using APWD-based detection of left atrial electrical arrhythmogenic remodeling) with subsequent AF screening (using AI-enabled single-lead-ECG as in ECG-watches), would yield higher diagnostic efficiency and allow to identify individuals at risk for AF and cardiovascular complications.

A large sample-sized study using AI-algorithm for AF prediction reported an AUC of 0.87 with overall accuracy of 79.4%, when using 10-s 12-lead-ECGs recorded during sinus rhythm (21). Although this AI-algorithm reaches similar diagnostic accuracies as our APWD-based models, the route-to-diagnosis remains unclear. In contrast to the AI-algorithm, our current APWD-based diagnostic models for AF have the strength in providing a comprehensible result by measurement of bi-atrial conduction time to detect individuals with underlying atrial arrhythmogenic substrate (10). Measurement of the PWD after digital recording and amplification enables physicians to diagnose atrial fibrotic cardiomyopathy. Therefore, the current methodology (APWD) can be considered as complementary to AI-based ECG-analysis.

## Limitations

The current cohort study demonstrates a high diagnostic potential for identification of individuals with *current* AF using the novel APWD models. Future large-scale longitudinal studies in population-based epidemiological cohorts are warranted to evaluate the diagnostic value of APWD-based models for prediction of *future* AF. Accurate measurement of p-wave duration necessitates digital 12-lead-ECGs that are recorded at a sampling rate 500–1,000 Hz, with acceptable signal-to-noise ratio (baseline noise should be below 0.08 mV) and amplified (60–120 mm/mV at 100 mm/s) with adequate visualization. Moreover, physicians need to be trained to correctly identify the onset and ending of the amplified p-waves, which should not be a major obstacle, as high expertise/training is also needed in many other diagnostic methods in medicine/medical imaging.

## Conclusion

The proposed APWD-based analysis detects the underlying atrial electrical abnormalities/substrate that predispose to AF development. Patients identified as high risk for AF (based on the proposed APWD-models), should undergo intensified ECG-monitoring for AF and may benefit from further diagnostics in search for underlying cardiovascular conditions that cause prolonged atrial conduction times and atrial cardiomyopathy.

## Data availability statement

The raw data supporting the conclusions of this article will be made available by the authors, without undue reservation.

## Ethics statement

This study was reviewed and approved by the institutional ethics committee of the University of Freiburg, Germany. Written informed consent was obtained from all participants for their participation in this study.

## Author contributions

TH, PS, BM-E, LM, ME, and NP collected the data. TH and PS measured the PWD in amplified and standard scale independently and blinded to patients’ rhythm status. TH and NP performed the statistical analyses. TH drafted the manuscript. PS, AJ, MB, HL, CA, MH, TA, DT, F-JN, and DW reviewed the manuscript. All authors approved submission of this manuscript.

## Footnotes

1
http://www.r-project.org/
