# Supplementary material for: Analysis of the amplified p-wave enables identification of patients with atrial fibrillation during sinus rhythm
Source: Front Cardiovasc Med. 2023 Feb 22;10:1095931. doi: 10.3389/fcvm.2023.1095931 (PMC9993657; doi:10.3389/fcvm.2023.1095931)
Supplement: Supplementary file 1 [file Data_Sheet_1.docx]

**Title:** Analysis of the amplified digitalized p-wave enables identification of patients with atrial fibrillation during sinus rhythm

**Authors:** Taiyuan Huang, Patrick Schurr, Bjoern Muller-Edenborn, Nicolas Pilia, Louisa Mayer,
Martin Eichenlaub, Juergen Allgeier, Marie Heidenreich, Christoph Ahlgrim, Marius Bohnen, Heiko Lehrmann, Dietmar Trenk, Franz-Josef Neumann, Dirk Westermann, Thomas Arentz and Amir Jadidi*

**Corresponding Author:**

Amir Jadidi, MD, PhD

University Heart Center Freiburg - Bad Krozingen

Suedring 15, 79189 Bad Krozingen, Germany

Tel. +49 76334334

[amir.jadidi@uniklinik-freiburg.de](mailto:amir.jadidi@uniklinik-freiburg.de)

**SupplementalAppendix**

**Supplemental tables (Page 2 - 6)**

**Supplemental figure (Page 7-12)**

**Supplemental tables**

**Supplemental Table 1.Baseline characteristics of cohorts from subtypes of rhythm**

| **Variables** | **Overall (n=1492)** | **Control (n=491)** | **AF (n=1001)** | **Paroxysmal AF (n=499)** | **Persistent AF (n=502)** | **P-value (AF vs Control)** | **P-value (Paroxysmal AF vs Control)** | **P-value (Persistent AF vs Control)** |
| --- | --- | --- | --- | --- | --- | --- | --- | --- |
| Age, years | 60.14 ± 14.42 | 60.02 ± 17.14) | 60.19 ± 12.88 | 59.25 ± 13.44 | 61.13 ± 12.25 | .828 | .401 | .225 |
| Female, n (%) | 631 (42.30%) | 262 (53.40%) | 369 (36.90%) | 165 (33.1%) | 204 (40.6%) | <.001 | <.001 | <.001 |
| BMI, kg/m2 | 27.09 ± 4.66 | 25.72 ± 4.39 | 27.75 ± 4.64 | 27.53 ± 4.62 | 27.97 ± 4.66 | <.001 | <.001 | <.001 |
| BSA, cm2 | 1.97 ± 0.23 | 1.87 ± 0.21 | 2.01 ± 0.22 | 2.01 ± 0.23 | 2.01 ± 0.22 | <.001 | <.001 | <.001 |
| LAD, mm | 40.19 ± 6.23 | 36.55 ± 4.95 | 41.90 ± 6.03 | 39.95 ± 5.19 | 43.80 ± 6.20 | <.001 | <.001 | <.001 |
| LVEF, % | 59.81 ± 9.87 | 61.67 ± 8.76 | 58.92 ± 10.25 | 61.18 ± 9.07 | 56.71 ± 10.84 | <.001 | .432 | <.001 |
| Hypertension, n (%) | 778 (52.10%) | 212 (43.20%) | 566 (56.5%) | 264 (52.9%) | 302 (60.2%) | <.001 | .002 | <.001 |
| Diabetes, n (%) | 127 (8.50%) | 35 (7.10%) | 92 (9.2%) | 45 (9.0%) | 47 (9.4%) | .180 | .275 | .201 |
| Stroke, n (%) | 36 (2.40%) | 4 (0.8%) | 32 (3.2%) | 11 (2.2%) | 21 (4.2%) | .004 | .073 | .001 |
| TIA, n (%) | 36 (2.40%) | 10 (2.0%) | 26 (2.6%) | 16 (3.2%) | 10 (2.0%) | .593 | .250 | .100 |
| CHD, n (%) | 177 (11.90%) | 35 (7.1%) | 142 (14.20%) | 68 (13.6%) | 74 (14.7%) | <.001 | .001 | <.001 |
| CHA2DS2-VASc score | 1.94 ± 1.45 | 1.74 ± 1.47 | 2.03 ± 1.43 | 1.80 ± 1.43 | 2.14 ± 1.43 | <.001 | .797 | <.001 |
| GFR (ml/min/1.73m2) | 80.58 ± 19.79 | 84.24 ± 19.71 | 78.79 ± 19.59 | 82.40 ± 18.08 | 75.20 ± 20.38 | <.001 | .135 | <.001 |
| creatinin clearance (mg/dl) | 0.96 ± 0.33 | 0.89 ± 0.21 | 0.99 ± 0.38 | 0.96 ± 0.37 | 1.03 ± 0.37 | <.001 | .001 | <.001 |
| Amiodarone, n (%) | 252 (16.90%) | NA | 252 (25.2%) | 74 (14.8%) | 178 (35.5%) | <.001 | <.001 | <.001 |
| Beta-blocker, n (%) | 800 (53.60%) | 152 (31.00%) | 648 (64.70%) | 319 (63.9%) | 329 (65.5%) | <.001 | <.001 | <.001 |

AF, atrial fibrillation; BMI, body mass index; BSA, body surface area; LAD, left atrial diameter; LVEF, left ventricular ejection fraction; TIA. Transient ischemic attack; CHD, coronary heart disease; GFR, glomerular filtration rate.

**Supplemental Table 2. Baseline characteristics of training and validation sets**

| **variables** | **Training set**  **(n = 896)** | **Validation set**  **(n = 596)** | **P value** |
| --- | --- | --- | --- |
| Age, years | 59.56 ± 14.91 | 61.01 ± 13.61 | .18 |
| Female, n (%) | 373 (41.6%) | 258 (43.3%) | .556 |
| Non-AF, n (%) | 297 (33.1%) | 194 (32.6%) | .85 |
| AF, n (%) | 599 (66.9%) | 402 (67.4%) | .85 |
| Paroxysmal AF, n (%) | 303 (33.8%) | 196 (32.9%) | .737 |
| Persistent AF, n (%) | 296 (33%) | 206 (34.6%) | .576 |
| BMI, kg/m2 | 27.04 ± 4.65 | 27.15 ± 4.67 | .56 |
| BSA, cm2 | 1.97 ± 0.23 | 1.96 ± 0.22 | .75 |
| LAD, mm | 40.27 ± 6.41 | 40.06 ± 5.96 | .52 |
| LVEF, % | 59.61 ± 10.01 | 60.11 ± 9.67 | .48 |
| Hypertension, n (%) | 463 (51.7%) | 315 (52.9%) | .672 |
| Diabetes, n (%) | 78 (8.7%) | 49 (8.2%) | .777 |
| Stroke, n (%) | 23 (2.6%) | 13 (2.2%) | .732 |
| TIA, n (%) | 19 (2.1%) | 17 (2.9%) | .392 |
| CHD, n (%) | 107 (11.9%) | 70 (11.7%) | .935 |
| CHA2DS2 VASc score | 1.94 ± 1.45 | 1.94 ± 1.46 | .90 |
| GFR, (ml/min/1.73m2) | 81 ± 19.99 | 79.95 ± 19.47 | .38 |
| Creatine, (mg/dl) | 0.96 ± 0.33 | 0.96 ± 0.34 | .66 |
| Amiodarone, n (%) | 157 (17.6%) | 95 (16.1%) | .480 |
| Beta blocker, n (%) | 469 (52.4%) | 331 (55.8%) | .203 |

AF, atrial fibrillation; BMI, body mass index; BSA, body surface area; LAD, left atrial diameter; LVEF, left ventricular ejection fraction; TIA. Transient ischemic attack; CHD, coronary heart disease; GFR, glomerular filtration rate.

**Supplemental Table3. Gender-specific subgroup analyses**

|  |  | **Training set** | | | **Validation set** | | |
| --- | --- | --- | --- | --- | --- | --- | --- |
|  | **Study Cohort** | **Control** | **AF** | **p value** | **Control** | **AF** | **p value** |
| Male subgroup | APWD | 126 ± 13 | 147 ± 22 | <.001 | 123 ± 12 | 150 ± 25 | <.001 |
|  | Standard PWD | 118 ± 10 | 120 ± 11 | .05 | 117 ± 10 | 120 ± 12 | .05 |
| Female subgroup | APWD | 118 ± 14 | 152 ± 22 | <.001 | 122 ± 15 | 151 ± 21 | <.001 |
|  | Standard PWD | 112 ± 11 | 123 ± 14 | <.001 | 115 ± 12 | 125 ± 14 | <.001 |

APWD, duration of amplified p-wave; AF, atrial fibrillation; PWD, p-wave duration

**Supplemental Table4. AUC of different models**

|  |  | **Training set** | | | **Validation set** | | |
| --- | --- | --- | --- | --- | --- | --- | --- |
| **Model** | **Component** | **AUC** | **95%CI** | | **AUC** | **95%CI** | |
| Reference | Standard PWD | 0.637 | 0.599 | 0.675 | 0.632 | 0.586 | 0.679 |
| Basic | APWD | 0.86 | 0.836 | 0.884 | 0.866 | 0.836 | 0.895 |
| PWI-based | APWD, aIAB, BSA | 0.892 | 0.868 | 0.916 | 0.885 | 0.856 | 0.915 |
| Integrated | APWD, aIAB, BSA, LAD | 0.916 | 0.897 | 0.936 | 0.902 | 0.874 | 0.928 |

AUC, area under the curve; CI, confidence interval;standard PWD, duration of standard (non-amplified) p-wave; APWD, duration of amplified p-wave; aIAB, advanced inter-atrial block; BSA, Body surface area; LAD, left atrial diameter.

**Supplemental Table5. Comparison for discriminative power between models**

|  | **training set (internal validation)** | | | | | **validation set (external validation)** | | | | |
| --- | --- | --- | --- | --- | --- | --- | --- | --- | --- | --- |
| Pairwise comparison | P value for AUC comparison | IDI | 95%CI | | p value | P value for AUC comparison | IDI | 95%CI | | p value |
| PWI-based model vs. Basic model | .019 | 0.041 | 0.030 | 0.052 | <0.001 | .018 | 0.067 | 0.047 | 0.086 | <0.001 |
| Integrated model vs. Basic model | <.001 | 0.051 | 0.038 | 0.064 | <0.001 | .009 | 0.070 | 0.049 | 0.090 | <0.001 |
| Integrated model vs. PWI-based model | <.001 | 0.010 | 0.003 | 0.017 | 0.006 | .005 | 0.003 | -0.003 | 0.008 | 0.293 |

AUC, area under the curve; IDI, integrated discrimination improvement; CI, confidence interval.

**Supplemental Fig. 1** Measurement of APWD at different scaling


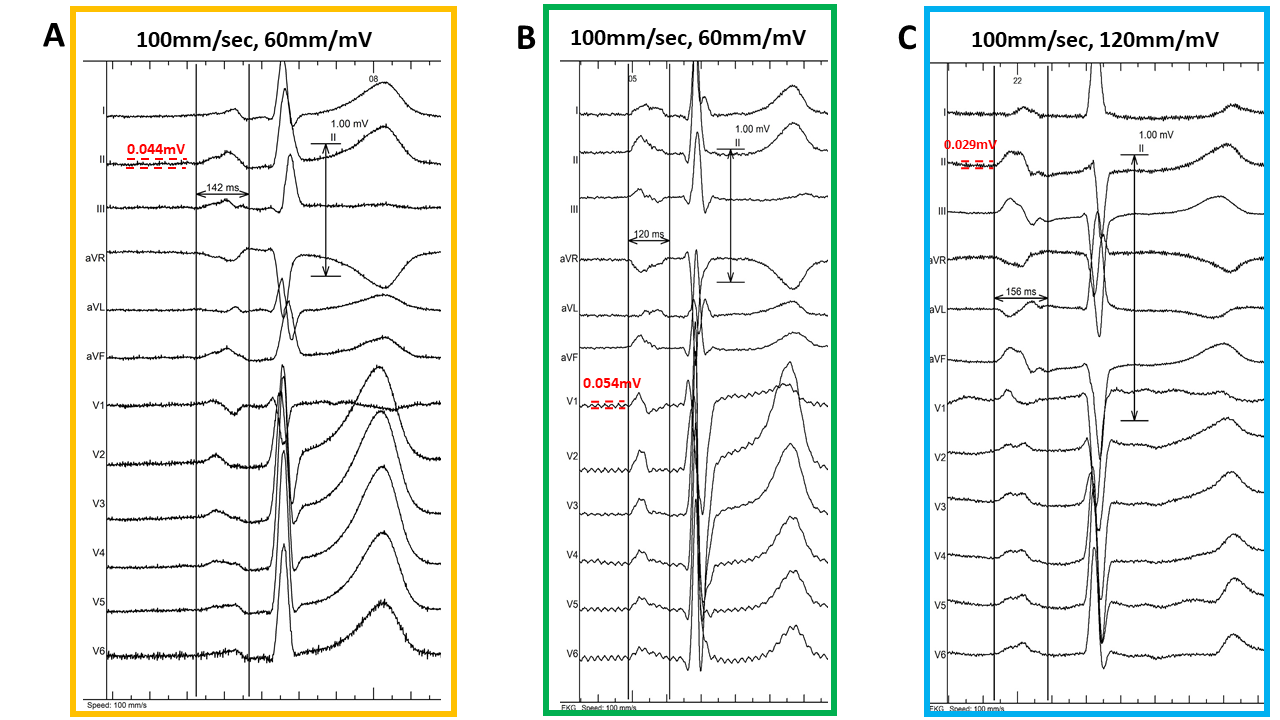


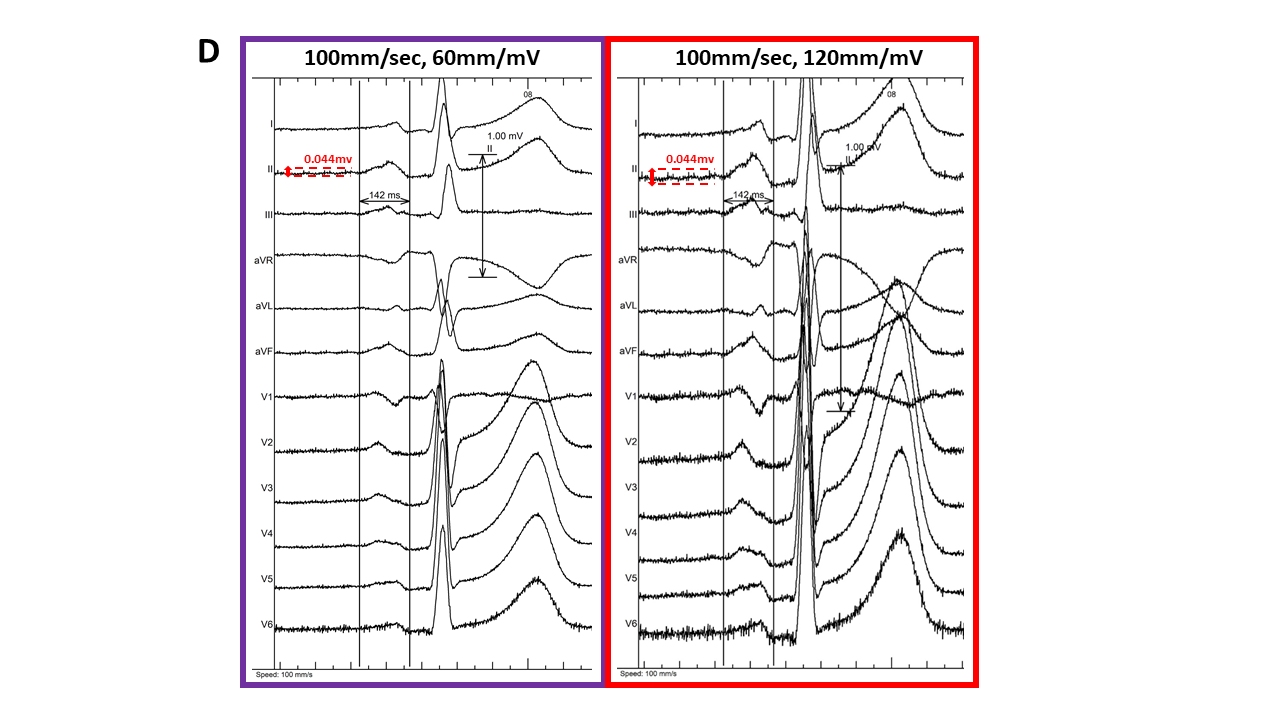


**Supplemental Fig. 1**ECGs of three different patients using two different amplified scaling are illustrated in panel A and B (100mm/second, 60mm/mV), panel C (100mm/second, 120mm/mV). Panel D illustrates the ECG of the same patient at two scaling (100mm/second, 60mm/mV in purple outline; 100mm/second, 120mm/mV in red outline) The noise level of each ECG is annotated in red dashed lines

**Supplemental Fig.2**Subgroup analyses in different genders


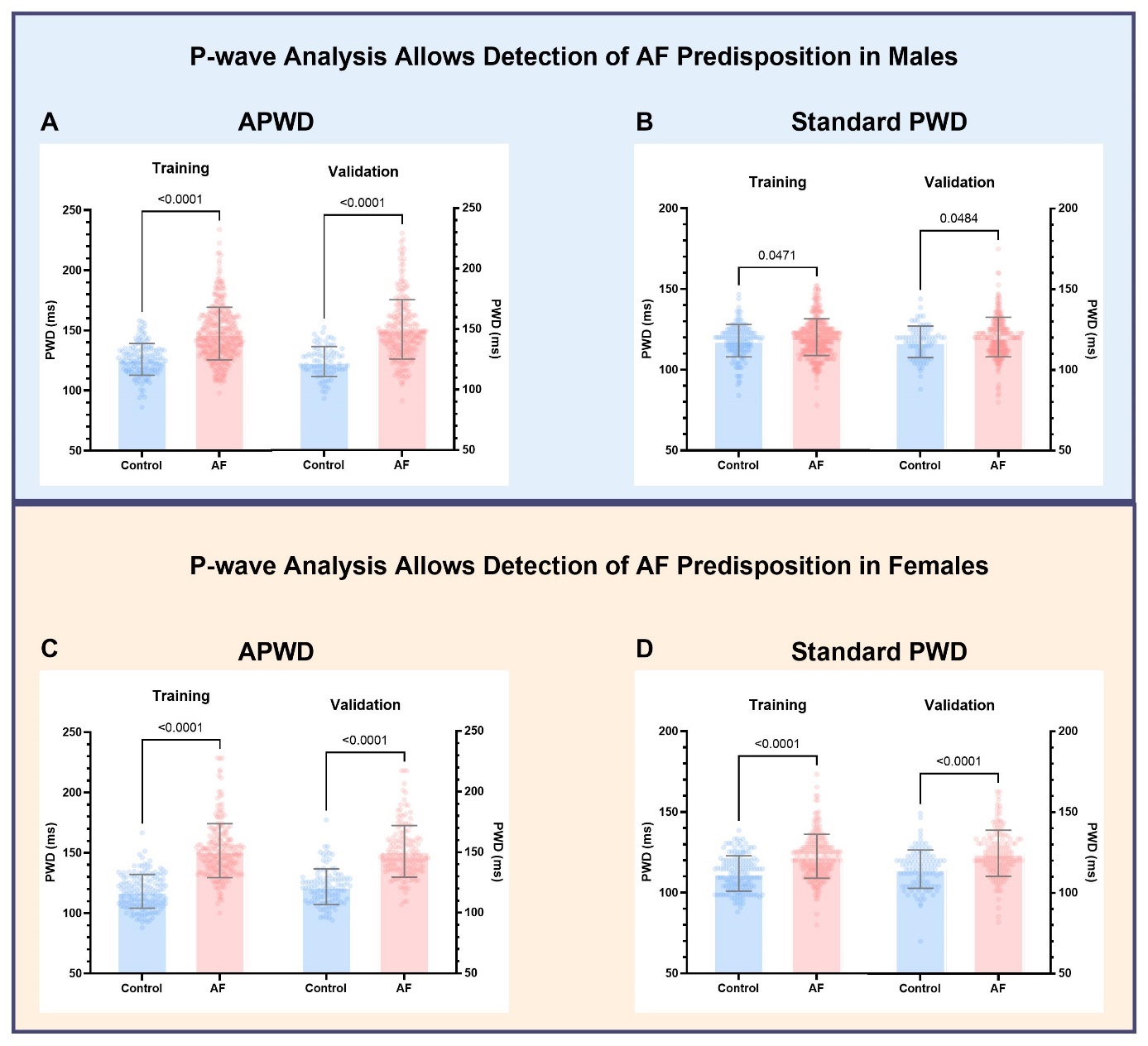


**Supplemental Fig.2**Subgroup results in male subgroup (upper panel) and female subgroup (lower panel). A and C represent the results using APWD in both training and validation sets. B and D illustrated the results using standard PWD. P value in each pairwise comparison is noted in each figure. *AF, atrial fibrillation; APWD*, duration of amplified p-wave; *Standard PWD*, duration of standard (non-amplified) p-wave

**Supplemental Fig.3** Reproducibility for APWD measurement


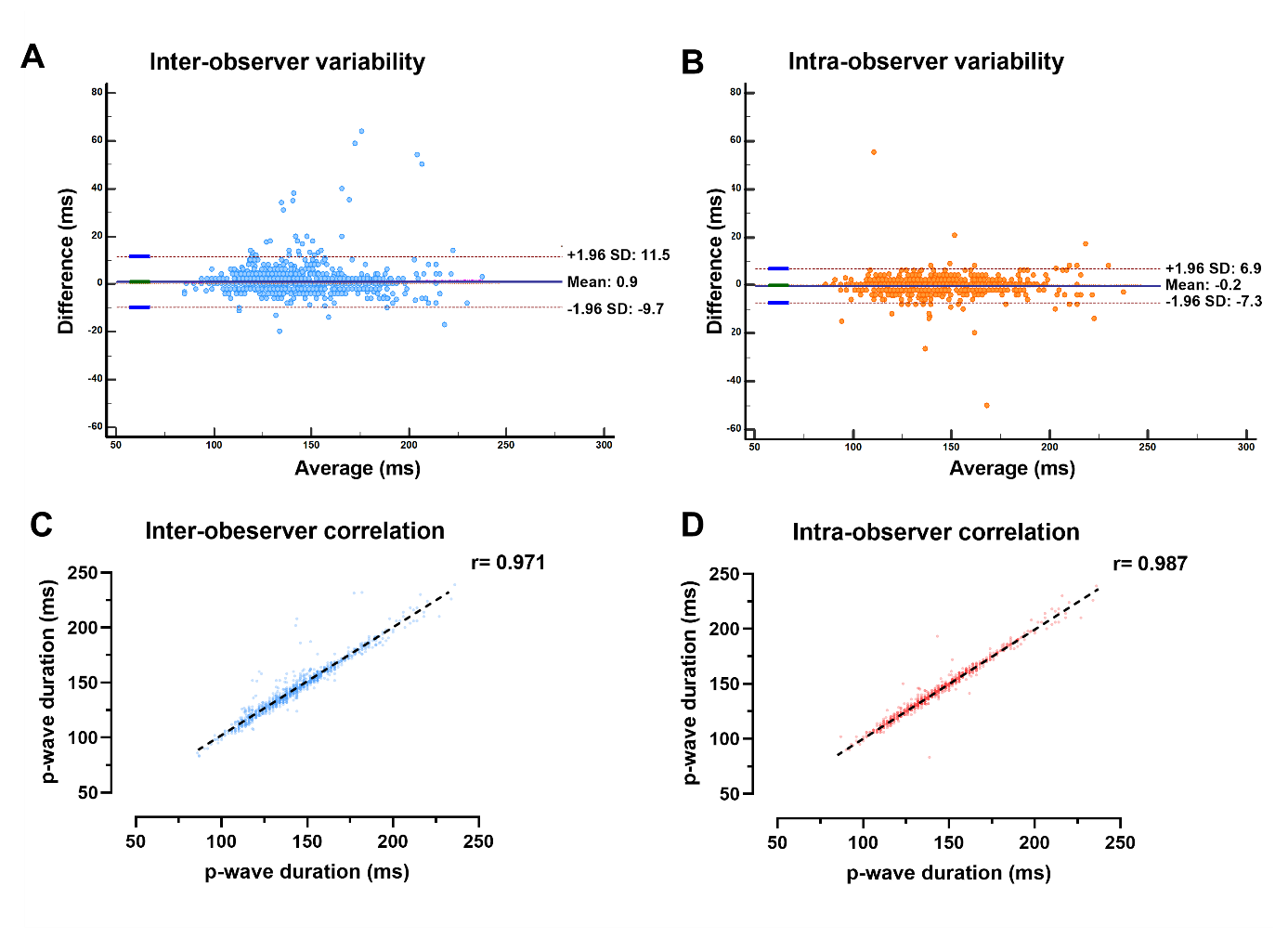


**Supplemental Fig.3**Reproducibility of APWD measurement.Inter- and intra-observer variability is illustrated using Bland-Altman Plots (A and B).In Panel A and B, the X-axis and Y-axis represent the average value and difference of APWD measured by two independent observers (inter-observer variability, blue) and the same observer in different dates (intra-observer variability, orange), respectively. The mean difference and 95% confidence interval of limits of agreement are described as the purple line and dashed lines, respectively. Correlation between APWD measurements of independent observers and within the same observer is illustrated using simple linear regression and fitted with a dashed line in Panel and D, respectively. Correlation coefficient (r) in each panel is annotated

**Supplemental Fig.4**Calibration curve


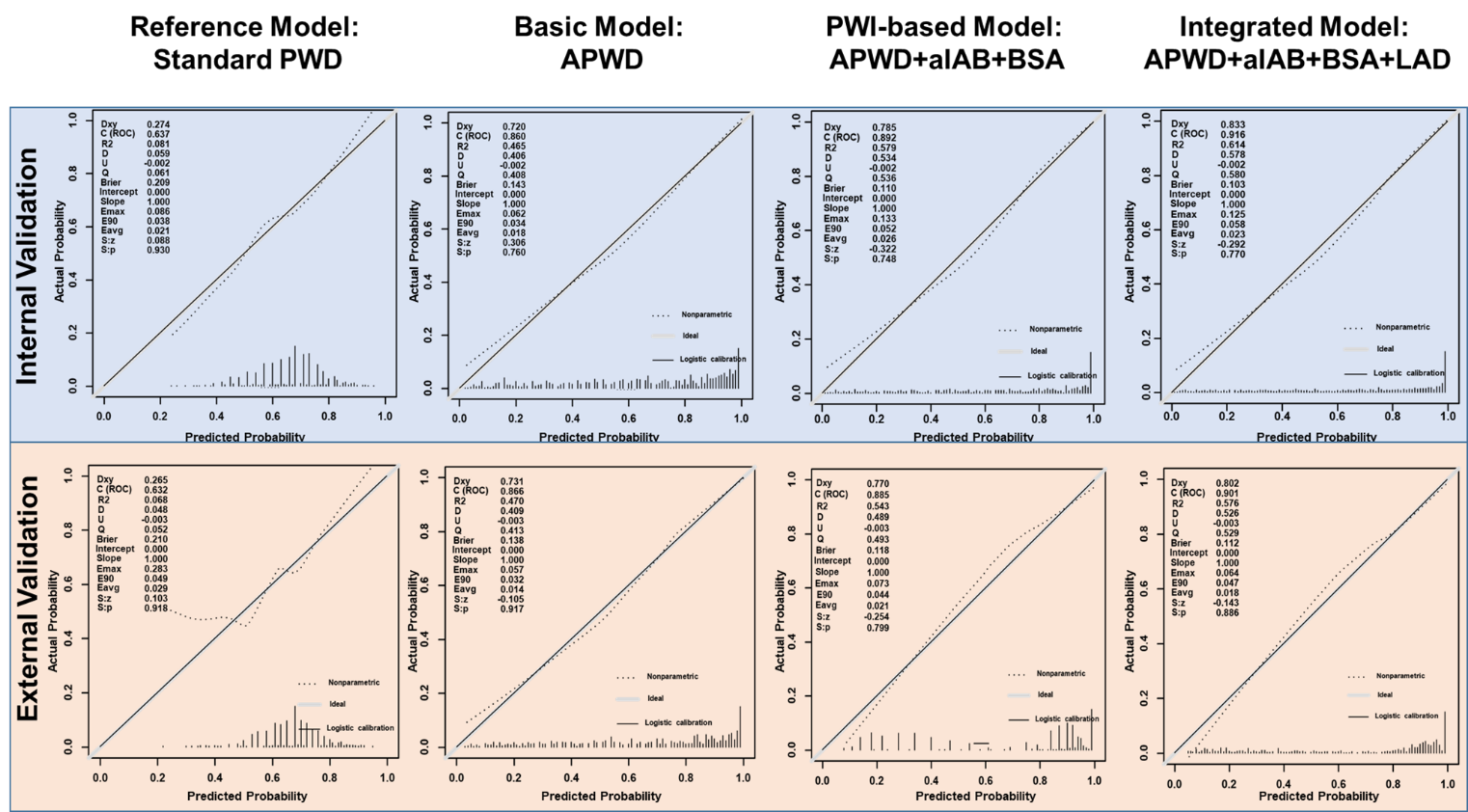


**Supplemental Fig. 4** Calibration curves of each model in internal (upper panel) and external validation (lower panel). The grey diagonal line “Ideal” represents the ideal diagnostic performance that the estimated probability equals the actual probability. The “Nonparametric” curve and “Logistic calibration” curve represent the actual and adjusted AF probability by each model, respectively

**Supplemental Fig.5**Diagnostic performance in internal and external validation


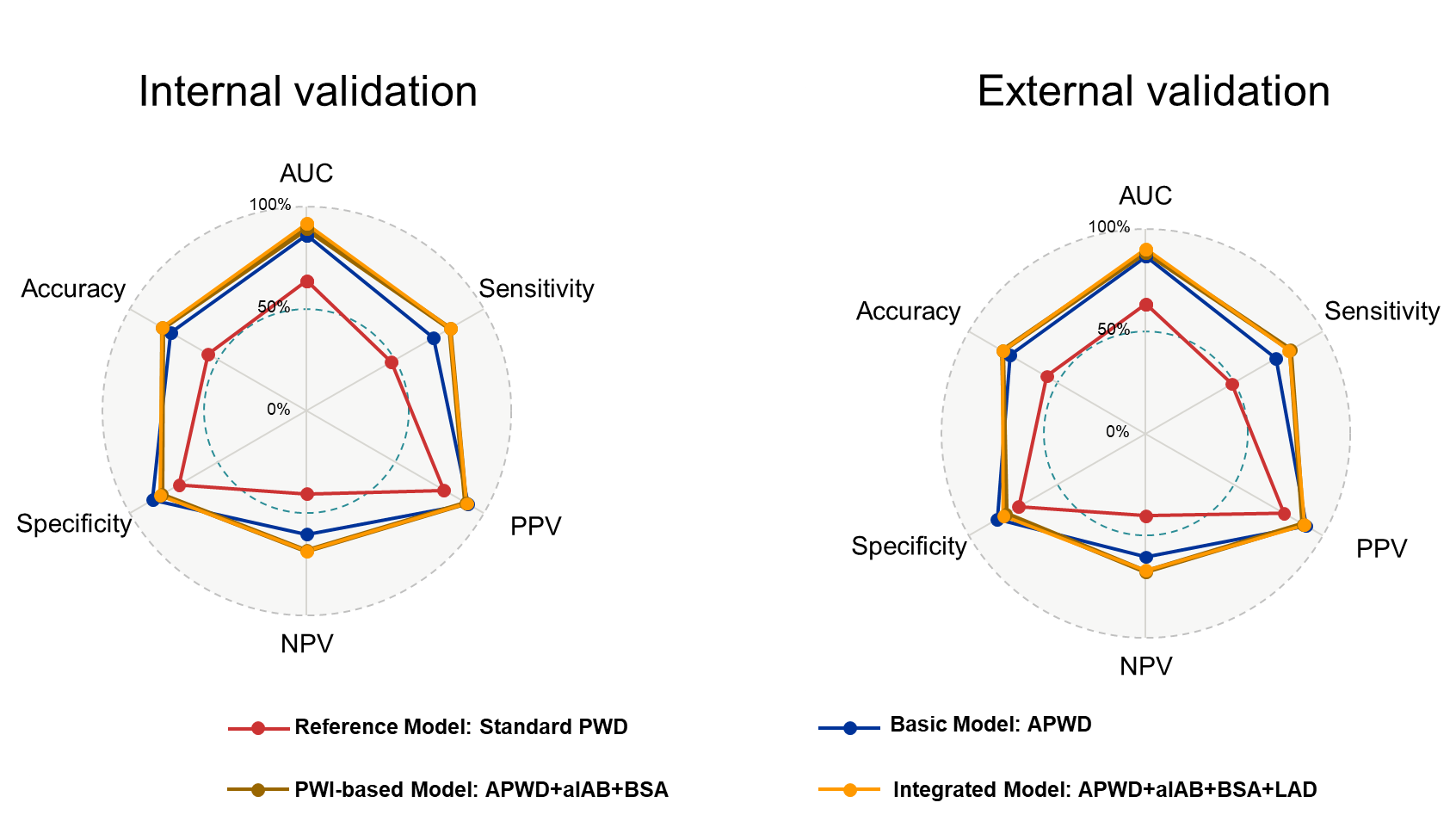


**Supplemental Fig. 5** Radar chart. Diagnostic performance of models in internal and external validation are evaluated in terms of accuracy, sensitivity, specificity, PPV and NPV using respective threshold: 121ms in Reference model, 136ms in Basic model, predicted AF-risk of 0.63 in PWI-based model and 0.65 in Integrated model. *AUC*, area under the curve; *PPV*, positive predictive value; *NPV*, negative predictive value

**Supplemental Fig.6**Illustrative examples of nomograms


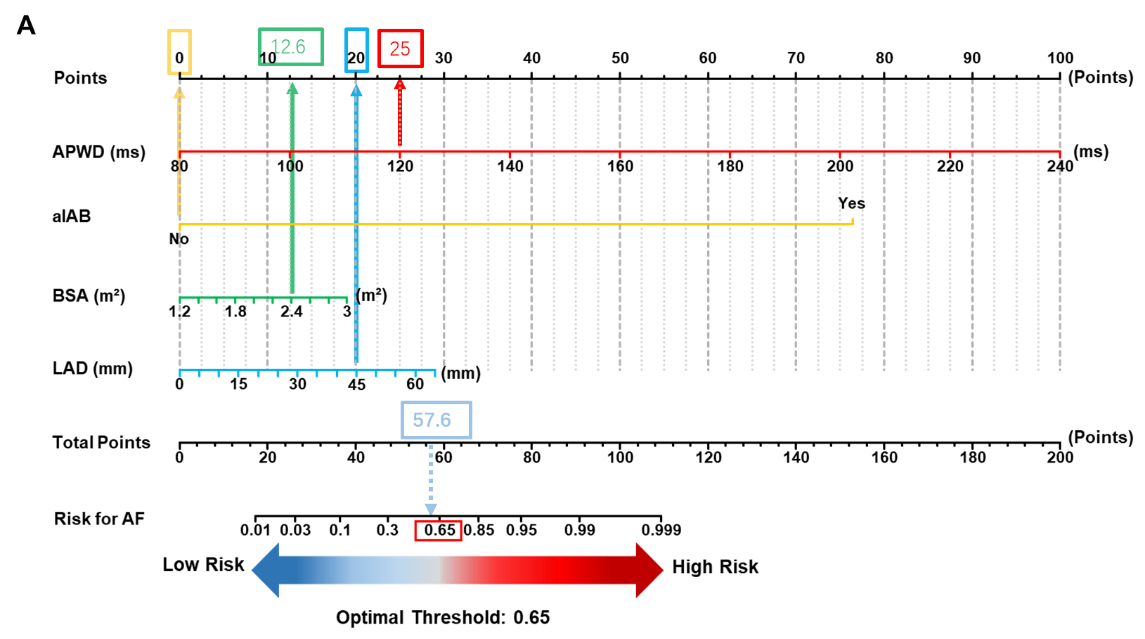


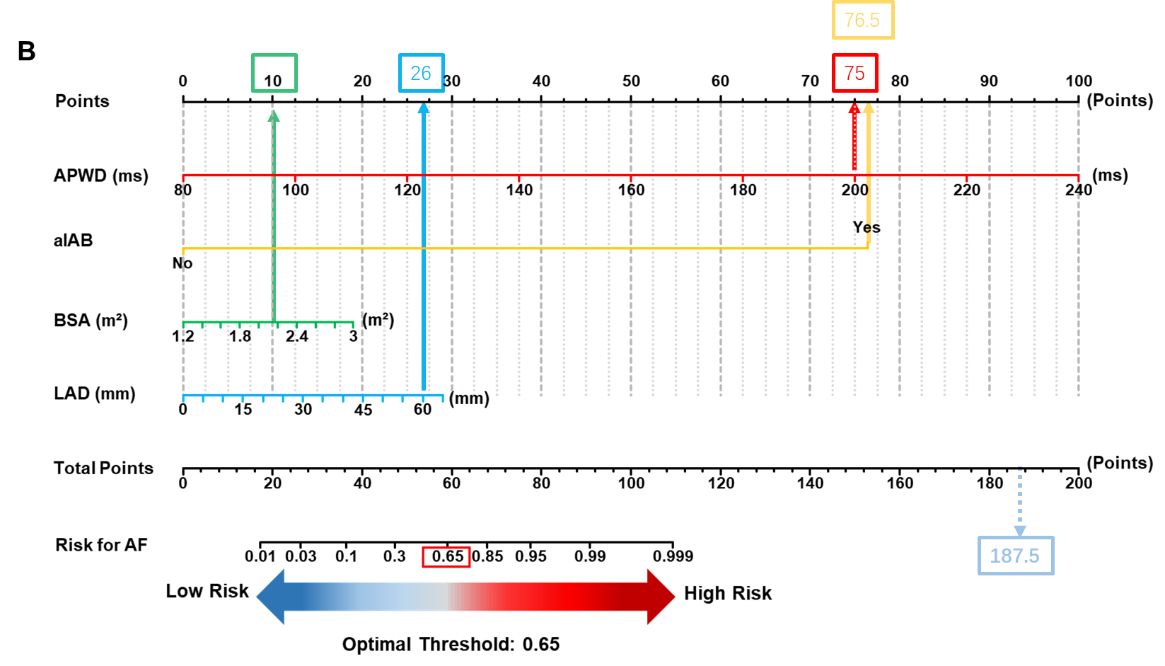


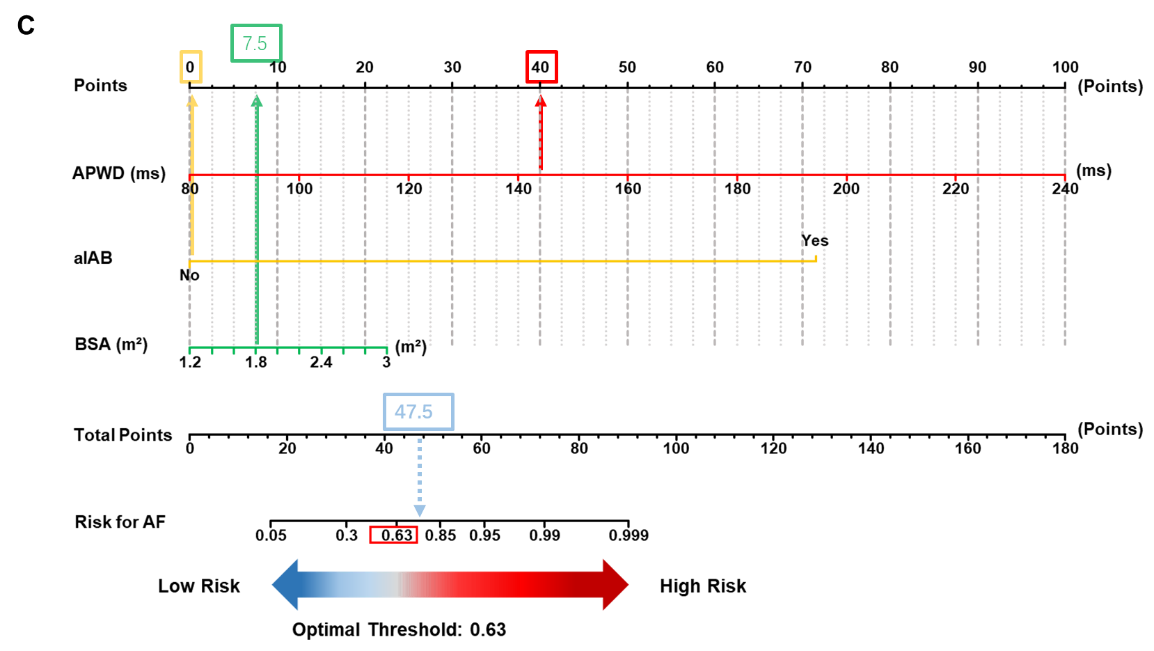


**Supplemental Fig.6**Illustration of using nomograms (integrated model in **panel A, B**and PWI-basedmodel in panel **C**) to estimate the risk for AF in three different individuals (A, B and C). **Panel A**, in an individual with APWD of 120 ms, absence of aIAB, BSA of 2.4m^2^ and LAD of 45mm. After corresponding each value with the point scale on the top (yellow, green, blue and red boxes), the total points of this individual would be a sum of 25 points (APWD 120ms) + 0 points (absence of aIAB) + 12.6 points (BSA 2.4 m^2^) + 20 points (LAD 45mm) which equaled to 57.6 points in the Total Points scale. By drawing a vertical line from the position of 57.6 points and extended it to the Risk for AF scale at the bottom, the estimated AF probability was below 0.65 (optimal threshold), thus identified as low risk for AF in this individual using integrated model nomogram. **Panel B**, in an individual with APWD of 200 ms (75 points, red box), presence of aIAB (approximately 76.5 points, yellow box), BSA of 2.15m^2^ (10 points, green box)and LAD of 60mm (approximately 26 points, blue box), the total sum of points would be 75+76.5+10+26=187.5 points, which far exceeds the upper limit (0.999) of risk scale, thus this individual would be estimated as high risk for AF by the integrated model nomogram. **Panel C**, in an individual with APWD of 144 ms, absence of aIAB and BSA of 1.8 m^2^. By corresponding the points assigned to each parameter in the PWI-based model nomogram, the sum of total points is 40 points (APWD 144ms) + 0 points (absence of aIAB) + 7.5 points (BSA 1.8 m^2^) = 47.5 points, which exceeds the threshold of 0.63 in the risk scale after drawing a vertical dashed line. Therefore, this individual is estimated as high risk for AF by the PWI-based model nomogram.
